# Cryo-EM of human Arp2/3 complexes provides structural insights into actin nucleation modulation by ARPC5 isoforms

**DOI:** 10.1242/bio.054304

**Published:** 2020-07-31

**Authors:** Ottilie von Loeffelholz, Andrew Purkiss, Luyan Cao, Svend Kjaer, Naoko Kogata, Guillaume Romet-Lemonne, Michael Way, Carolyn A. Moores

**Affiliations:** 1 Department of Biological Sciences, Institute of Structural and Molecular Biology, Birkbeck College, London WC1E 7HX, UK; 2 The Francis Crick Institute, 1 Midland Road, London NW1 1AT, UK; 3 Université de Paris, CNRS, Institut Jacques Monod, 75013 Paris, France; 4 Department of Infectious Disease, Imperial College London, London W2 1PG, UK

**Keywords:** Arp2/3, Actin, Cytoskeleton, Cryo-EM, Isoforms, Nucleation

## Abstract

The Arp2/3 complex regulates many cellular processes by stimulating formation of branched actin filament networks. Because three of its seven subunits exist as two different isoforms, mammals produce a family of Arp2/3 complexes with different properties that may be suited to different physiological contexts. To shed light on how isoform diversification affects Arp2/3 function, we determined a 4.2 Å resolution cryo-EM structure of the most active human Arp2/3 complex containing ARPC1B and ARPC5L, and compared it with the structure of the least active ARPC1A-ARPC5-containing complex. The architecture of each isoform-specific Arp2/3 complex is the same. Strikingly, however, the N-terminal half of ARPC5L is partially disordered compared to ARPC5, suggesting that this region of ARPC5/ARPC5L is an important determinant of complex activity. Confirming this idea, the nucleation activity of Arp2/3 complexes containing hybrid ARPC5/ARPC5L subunits is higher when the ARPC5L N-terminus is present, thereby providing insight into activity differences between the different Arp2/3 complexes.

## Abstract (precis)

**Summary:** The Arp2/3 complex stimulates formation of branched actin filament networks and exhibits isoform diversity in mammals. We show how different Arp2/3 subunit isoforms contribute to differences in complex function.

## INTRODUCTION

The Arp2/3 complex is evolutionarily conserved and built from two actin related proteins (Arp2 and Arp3) and five other protein subunits (ARPC1-5) (Molinie and Gautreau, 2018; Goley and Welch, 2006). It plays an essential role in many cellular processes, most notably cell migration (Goley and Welch, 2006; Krause and Gautreau, 2014), and also has newly identified roles in DNA repair (Caridi et al., 2018; Schrank et al., 2018). When activated by a nucleation-promoting factor such as WAVE or WASP, the Arp2/3 complex is unique in its ability to assemble branched actin networks by stimulating new filament growth from the side of existing actin filaments (Campellone and Welch, 2010).

Until recently, the Arp2/3 complex has been considered as a single entity. However, in mammals, Arp3, ARPC1 and ARPC5 are present as two isoforms – Arp3, Arp3B, ARPC1A, ARPC1B, ARPC5 and ARPC5L – that share 91, 67 and 67% sequence identity, respectively (Abella et al., 2016; Pizarro-Cerdá et al., 2017). This raises the question as to whether different Arp2/3 complexes have evolved unique properties that are adapted to their particular cellular, developmental or physiological roles. Recent work has shown that the ARPC1 and ARPC5 isoforms differentially affect the actin nucleating properties of the Arp2/3 complex and the stability of the branched filament networks it generates (Abella et al., 2016). Furthermore, tissue-specific expression patterns of subunit isoforms, together with isoform-specific susceptibility to disease-causing point mutations, point to distinct physiological roles for particular Arp2/3 isoforms in nuclei positioning in skeletal muscle (Roman et al., 2017), as well as cytotoxic T lymphocyte maintenance and activity (Brigida et al., 2018; Kuijpers et al., 2017; Randzavola et al., 2019; Somech et al., 2017; Volpi et al., 2019; Roman et al., 2017; Kahr et al., 2017).

Currently, the available high-resolution structures of mammalian Arp2/3 cannot address the role of isoform-specified diversity. Using natively purified proteins, only a single isoform combination – ARPC1B-ARPC5 – of mammalian Arp2/3 has been visualised (for example, the first structure described by Robinson et al., 2001); this combination was shown to have intermediate nucleation activity (Abella et al., 2016). To begin to understand how subunit composition affects the properties of human Arp2/3 complexes, we used recombinant protein expression and cryo-electron microscopy (cryo-EM) to determine the structure of the most active human Arp2/3 complex, containing ARPC1B and ARPC5L subunits, referred to here as Arp2/3-C1B-C5L. We compared it with the structure of a complex containing ARPC1A and ARPC5 (Arp2/3-C1A-C5), which has the lowest activity (Abella et al., 2016). Our structures show isoform-specific differences in the N-terminus of ARPC5/5L and suggest that these structural variations mediate different activities. Using protein engineering, we show that inclusion of the N-terminus of ARPC5L in hybrid Arp2/3 complexes enhances their actin nucleation activity, thereby showing how different Arp2/3 subunit isoforms contribute to differences in complex activation and function.

## RESULTS AND DISCUSSION

### Overview of human Arp2/3 complex structure

**Fig. 1.**
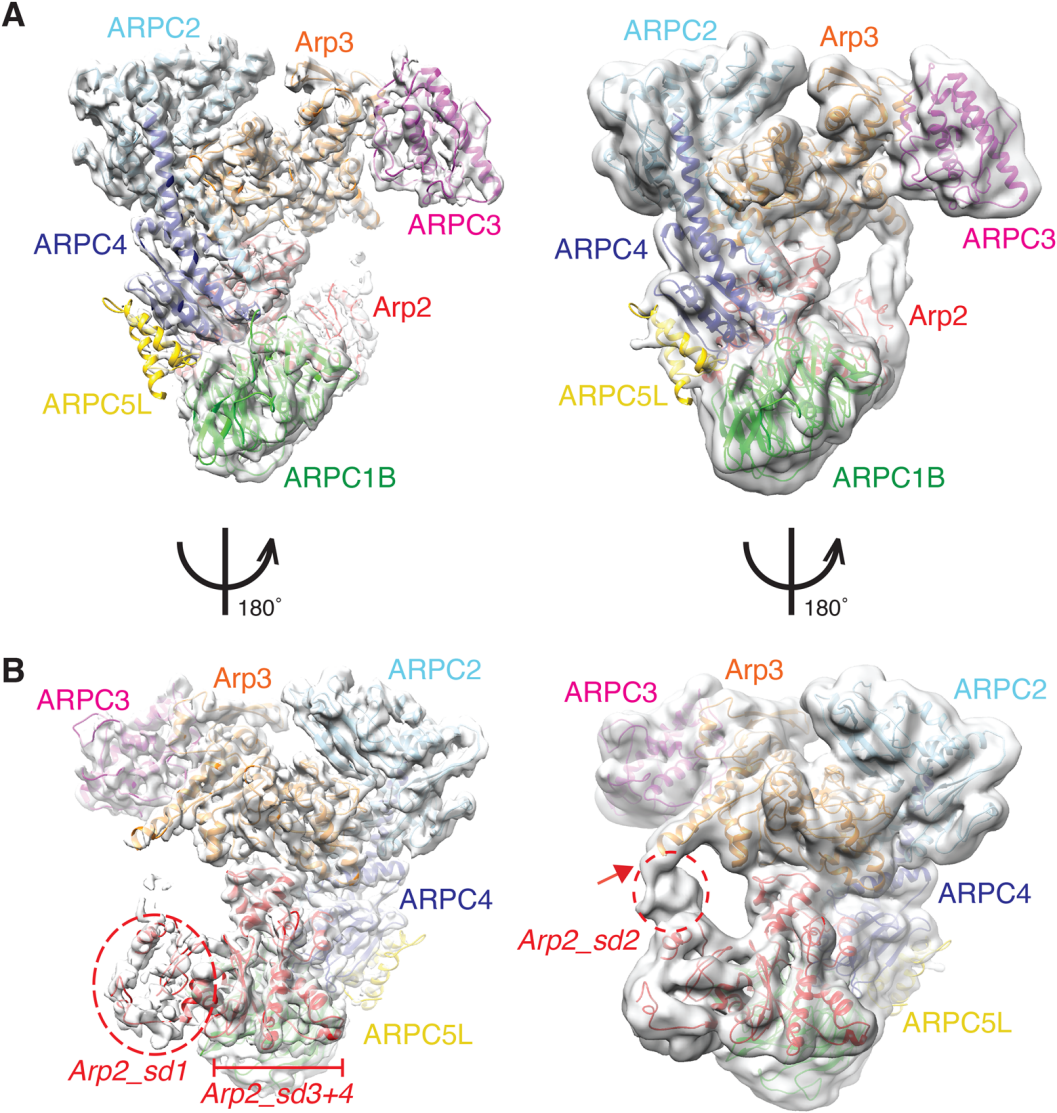
**The cryo-EM structure of the human Arp2/3 ARPC1B-ARPC5L complex.** (A) Left, overview of the cryo-EM reconstruction of Arp2/3-C1B-C5L with the docked model in the density, viewed and coloured as originally presented by Robinson et al. (2001): Arp2: red; Arp3: orange; ARPC1B: green; ARPC2: cyan; ARPC3: magenta; ARPC4: light blue; ARPC5L: yellow; right, same view of the reconstruction with a ∼8 Å low-pass filter applied showing more flexible regions of the complex at lower resolution; (B) left, 180° rotated view compared to A of the Arp2/3-C1B-C5L reconstruction and model; right, same view of the reconstruction with a ∼8 Å low-pass filter applied showing more flexible regions of the complex at lower resolution, which includes flexible connectivity between Arp2 and Arp3 (red arrow) and parts of ARPC5L. sd, subdomains of Arp2.

We first determined the 4.2 Å resolution structure and calculated a pseudo-atomic model of the most active human Arp2/3 complex Arp2/3-C1B-C5L (Fig. 1, Table 1; Fig. S1A–C) (Abella et al., 2016). The complex shows the characteristic triangle shape – ∼150 Å×130 Å and ∼100 Å thick – seen in previous crystallographically determined structures of Arp2/3 complex purified from bovine brain and yeast (Robinson et al., 2001; Nolen and Pollard, 2007, 2008; Nolen et al., 2009). As observed in these previous structures, the intertwined ARPC2/4 subunits (Fig. 1A,B, light/dark blue) form the platform for complex assembly, with Arp3/ARPC3 (Fig. 1A,B, orange/magenta) and Arp2/ARPC1B/ARPC5L (Fig. 1A,B, red/green/yellow) constituting distinct protrusions. Apart from a few residues at the N- and C-termini, the ARPC1B, C2, C3 and C4 subunits are completely visualised. Similarly, Arp3 is almost completely represented in the cryo-EM density. In contrast, while subdomains 3 and 4 of Arp2 are well ordered and at a similar resolution to the rest of the reconstruction, density corresponding to subdomain 1 is present but poorly defined and subdomain 2 is even less distinct (Fig. 1B). In addition, although density corresponding to the 2 C-terminal α-helices of ARP5CL lies adjacent to ARPC4, there is a sharp fall-off in the strength of the cryo-EM density for the rest of the subunit further from the body of the complex (described in detail below). The regions of different flexibility within the complex also manifest in the variations in local resolution of the reconstruction (Fig. S1D).

**Table 1.**
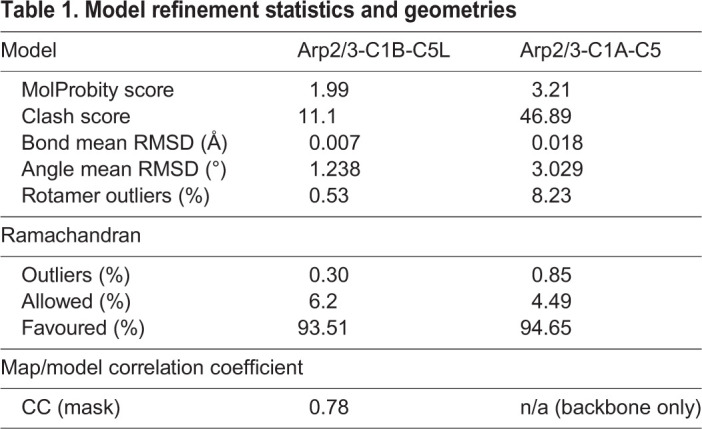
**Model refinement statistics and geometries**

### Conformation of ATP-bound Arp3 and Arp2

**Fig. 2.**
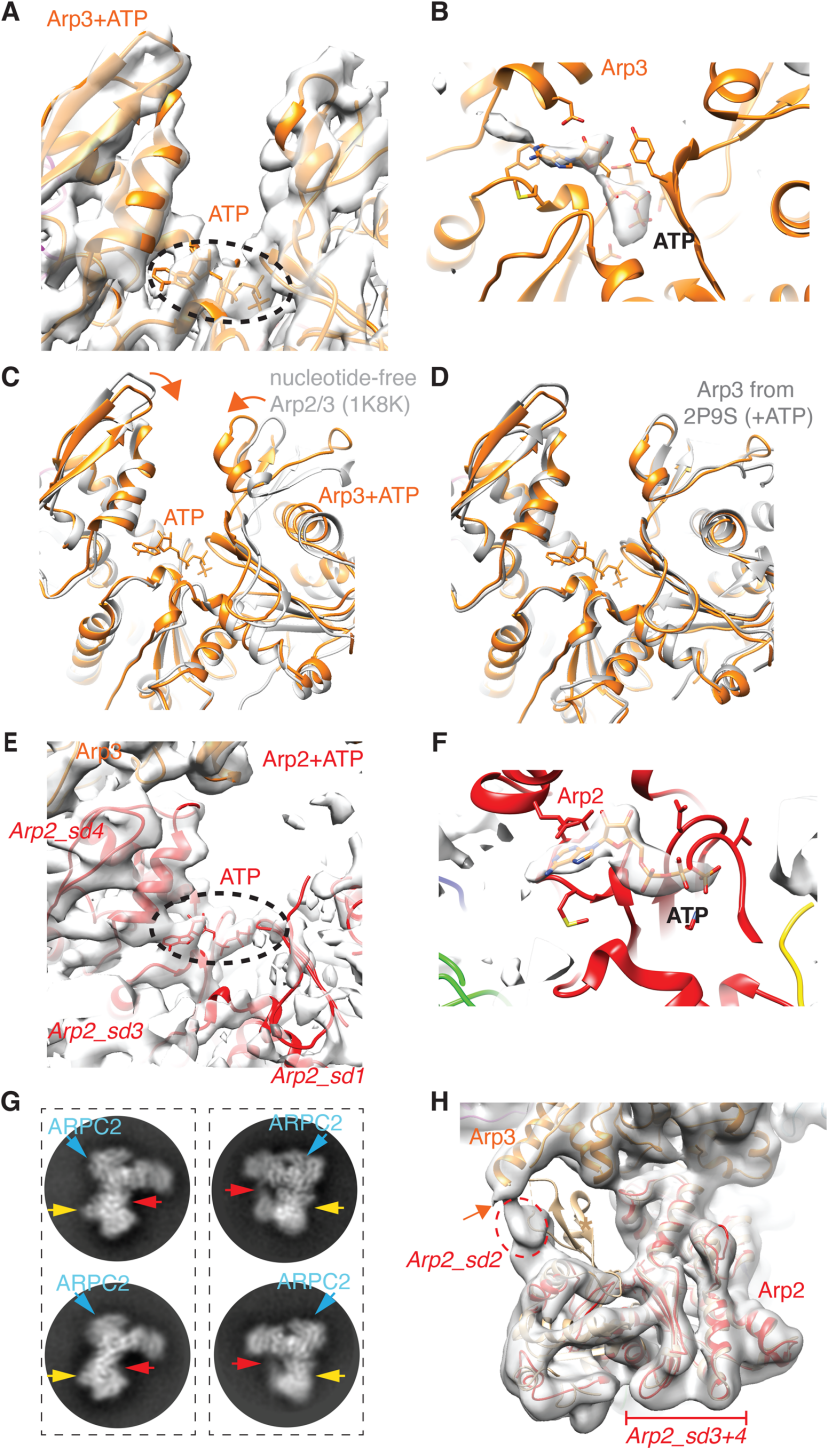
**Nucleotide binding sites of Arp3 and Arp2 in Arp2/3-C1B-C5.** (A) Cryo-EM reconstruction and model of nucleotide binding pocket of Arp3 with density corresponding to bound ATP indicated (dotted black oval); (B) ribbon depiction of the Arp3 model with density corresponding to bound nucleotide shown in surface representation. This density is the calculated difference between our cryo-EM reconstruction and simulated density of the atomic model without nucleotide at equivalent resolution, calculated using Chimera. This supports the conclusion that ATP is bound to Arp3; (C) conformation of the nucleotide binding pocket of ATP-bound Arp3 in Arp2/3-C1B-C5L aligned (on subdomain 3) with a previously determined structure of nucleotide-free Arp2/3 (PDB 1K8K), showing closure of the pocket in the presence of bound nucleotide; (D) conformation of the nucleotide binding pocket of ATP-bound Arp3 aligned (on subdomain 3) with a previously determined structure of ATP-bound Arp2/3 (2P9S; Nolen and Pollard, 2007), showing equivalent closure of the pocket in the presence of bound nucleotide compared to the absence of nucleotide; (E) cryo-EM reconstruction and model of nucleotide binding pocket of Arp2 with visible subdomain regions labelled and density corresponding to bound ATP indicated (dotted black oval); (F) ribbon depiction of the Arp2 model with density corresponding to bound nucleotide shown in surface representation. This density is the calculated difference between our cryo-EM reconstruction and simulated density of the atomic model without nucleotide at equivalent resolution, calculated using Chimera. This supports the conclusion that ATP is bound to Arp2; (G) 2D class averages of Arp2/3-C1B-C5L showing views corresponding to Fig. 1A (left panels) and Fig. 1B (right panels) illustrating the variable density corresponding to subdomain 2 of Arp2 (red arrows) and to ARPC5L (yellow arrows). ARPC2 is also labelled for reference (blue arrows). (H) Arp2 in the GMF-inhibited Arp2/3 (PDB 4JD2, in tan) (Luan and Nolen, 2013) is shown aligned with Arp2 (red) in Arp2/3-C1B-C5L within the low-pass filtered cryo-EM density. As previously shown, in the Arp2/3-C1B-C5L reconstruction Arp2 subdomain 2 is flexibly connected to Arp3 helix-α9 (orange arrow), whereas the well-defined structure of the Arp2 subdomain 2 in the GMF-inhibited structure adopts a different conformation which protrudes from the EM density (tan asterisk). For clarity, other subunits within the GMF-inhibited complex are not shown. sd, subdomains of Arp2.

ATP is a key regulator of Arp2/3 complex activity (Le Clainche et al., 2001; Nolen and Pollard, 2007; Ingerman et al., 2013; Rodnick-Smith et al., 2016; Martin et al., 2006), and density corresponding to ATP, assigned based on comparison with previously determined structures, is bound to Arp3 in the Arp2/3-C1B-C5L reconstruction (Fig. 2A,B). Consistent with this, the Arp3 nucleotide binding pocket adopts a closed conformation as defined previously (Nolen and Pollard, 2007), such that the outer portions subdomains 2 and 4 of Arp3 are closer together than in structures with no bound nucleotide (e.g. 1K8K; Fig. 2C). Furthermore, the conformation of human Arp3 in our reconstruction is similar to that of previously determined ATP-bound bovine Arp3 in this region (Fig. 2D).

In Arp2, density corresponding to bound nucleotide, again assigned based on comparison with previously determined structures, is present at the junction of subdomains 3 and 4 (Fig. 2E,F). Density from Arp2 subdomain1 exhibits some ordering immediately adjacent to the nucleotide density, consistent with the role of this region in nucleotide binding, whereas only low-resolution density is present for most of subdomains 1 and 2, and is of insufficient quality for models to be calculated. The overall weak 3D density of this region of Arp2 is consistent with the conformational variability seen in 2D classes (Fig. 2G), and with the flexibility that has been previously observed in Arp2/3 crystal structures (Robinson et al., 2001; Nolen and Pollard, 2007, 2008; Nolen et al., 2009). These data all support the idea that flexibility is intrinsic to this region of the Arp2/3 complex in the presence of ATP and that ATP binding does not induce large-scale conformational changes in the complex (Espinoza-Sanchez et al., 2018), in contrast to the large ATP-induced rearrangements previously proposed by Rodnick-Smith et al. (2016). Earlier low-resolution EM characterizations of Arp2/3 probably also captured the dynamic properties of this region rather than activated states of the complex (Rodal et al., 2005; Sokolova et al., 2017). It is also possible that interaction with the grid surface in negative stain experiments may cause additional ordering of Arp2 subdomains 1 and 2 (Espinoza-Sanchez et al., 2018), in contrast to what we observe in our cryo-EM experiments.

The previously determined structure of inhibitor-bound Arp2/3 provides the most complete structural view of Arp2 (Luan and Nolen, 2013) in which subdomain 2 of Arp2 lies close to the loop connecting helix-α7 and -α8 in Arp3. Interestingly, although the Arp2 density in our uninhibited complex is relatively weak, the connectivity with Arp3 is not the same as in the inhibited structure, but rather occurs via helix-α9 (Fig. 2H). This supports the idea that Arp2/3 complex inhibition operates at least in part by restricting the movements of otherwise flexible regions of Arp2 and implies that flexibility in this region is important for subsequent steps in its activation. The connectivity between Arp2 and Arp3 in our reconstruction may represent a route of allosteric communication that is part of the activation process for the complex, a route that is blocked by inhibitor binding. Evidence of such allosteric communication between Arp2 and Arp3 is also provided by the recent cryo-EM reconstruction of NPF-bound human Arp2/3-C1B-C5 complex (Zimmet et al., 2020), in which subdomain 2 of Arp2, while visible, remains one of the most flexible parts of the structure.

**Fig. 3.**
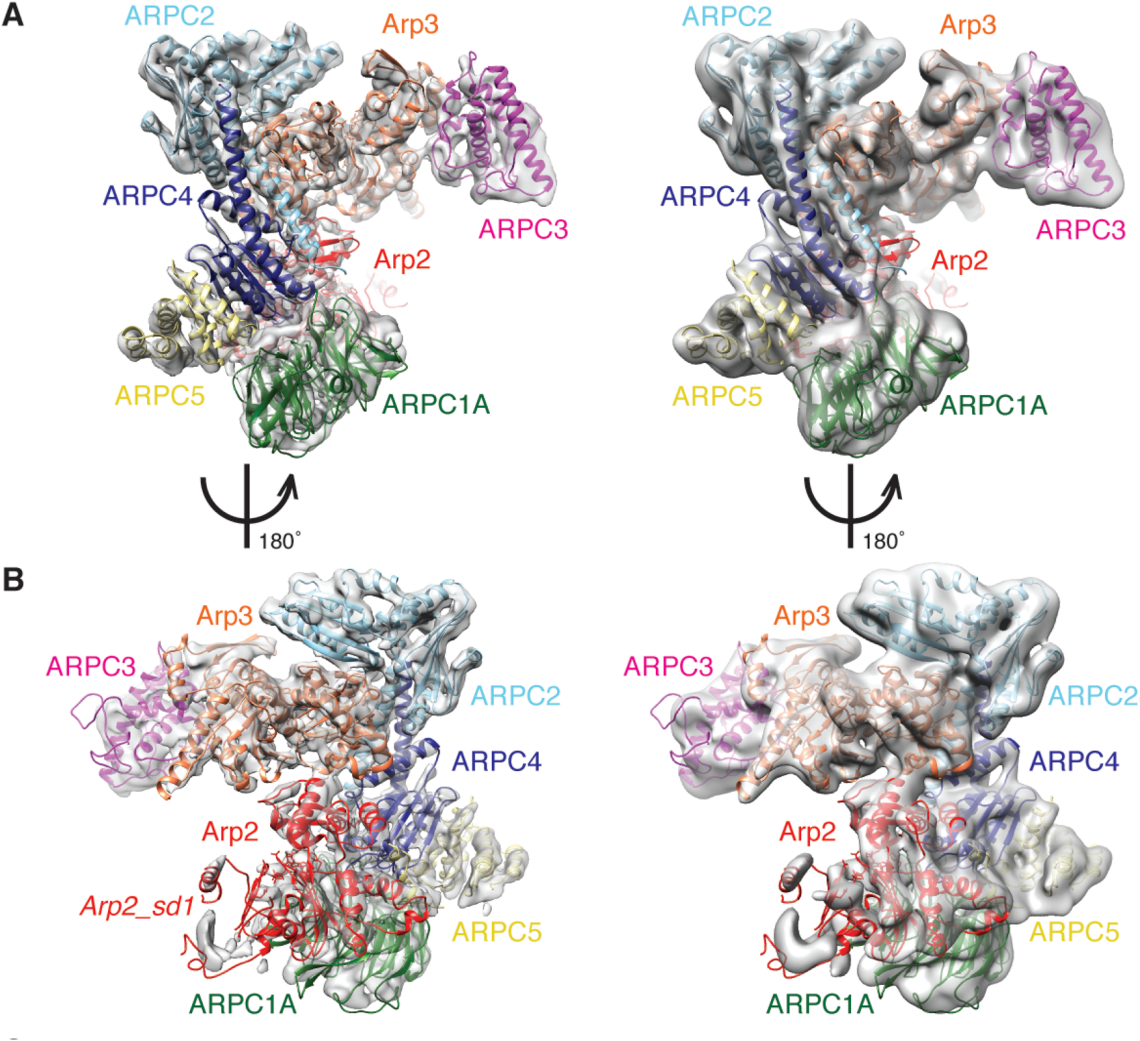
**The cryo-EM structure of the human Arp2/3-ARPC1A-ARPC5 complex.** (A) Left, cryo-EM reconstruction of Arp2/3-C1A-C5; right, same view with a ∼8 Å low-pass filter applied to potentially reveal more flexible regions of the complex at lower resolution. The docked model is coloured as in previous figures: Arp2: red; Arp3: orange; ARPC2: cyan; ARPC3: dark pink; ARPC4: blue, except that ARPC1A is dark green and ARPC5 is pale yellow; (B) left, 180° rotated view compared to (A) of the Arp2/3-C1A-C5 reconstruction and model; right, same view of the reconstruction with a ∼8 Å low-pass filter applied. sd, subdomains of Arp2.

To investigate how general these observations are for cryo-EM-determined reconstructions of Arp2/3 complexes, we also calculated the 3D reconstruction of human Arp2/3 complex containing ARPC1A and ARPC5 subunits (Fig. 3). This reconstruction has a slightly lower overall resolution (4.5 Å; Fig. S2A), its constituent particles also exhibit non-isotropic angular orientations (Fig. S2B) and the reconstruction shows non-isotropic variation in local resolution (Fig. S2C). However, it is clear from this structure that the overall conformation of the Arp2/3-C1A-C5 reconstruction is extremely similar to that of Arp2/3-C1B-C5L, apart from the density corresponding to ARPC5/C5L (see below). In particular, in each complex Arp2 and Arp3 are similarly organised with respect to each other, although subdomains 1 and 2 in Arp2 are even more flexible in the Arp2/3-C1A-C5 structure compared to Arp2/3-C1B-C5L (Fig. 3A,B and Fig. S2D with Figs 1A,B and 2G). This supports the idea that subdomains 1 and 2 of Arp2 are intrinsically flexible even in the presence of activating nucleotide, and that this flexibility is important for subsequent activation steps in the presence of additional ligands. The overall conformational similarity of these two isoform-specific complexes – which have been shown to have very different actin assembly promoting activities both *in vitro* and in cells (Abella et al., 2016) – implies that the isoform-specific differences observed are not mediated by fundamentally different architectures of the complex, but rather by alternative regulatory effects conferred by isoform-specific residues.

### Isoform specific subunit conformation and determinants of activity

**Fig. 4.**
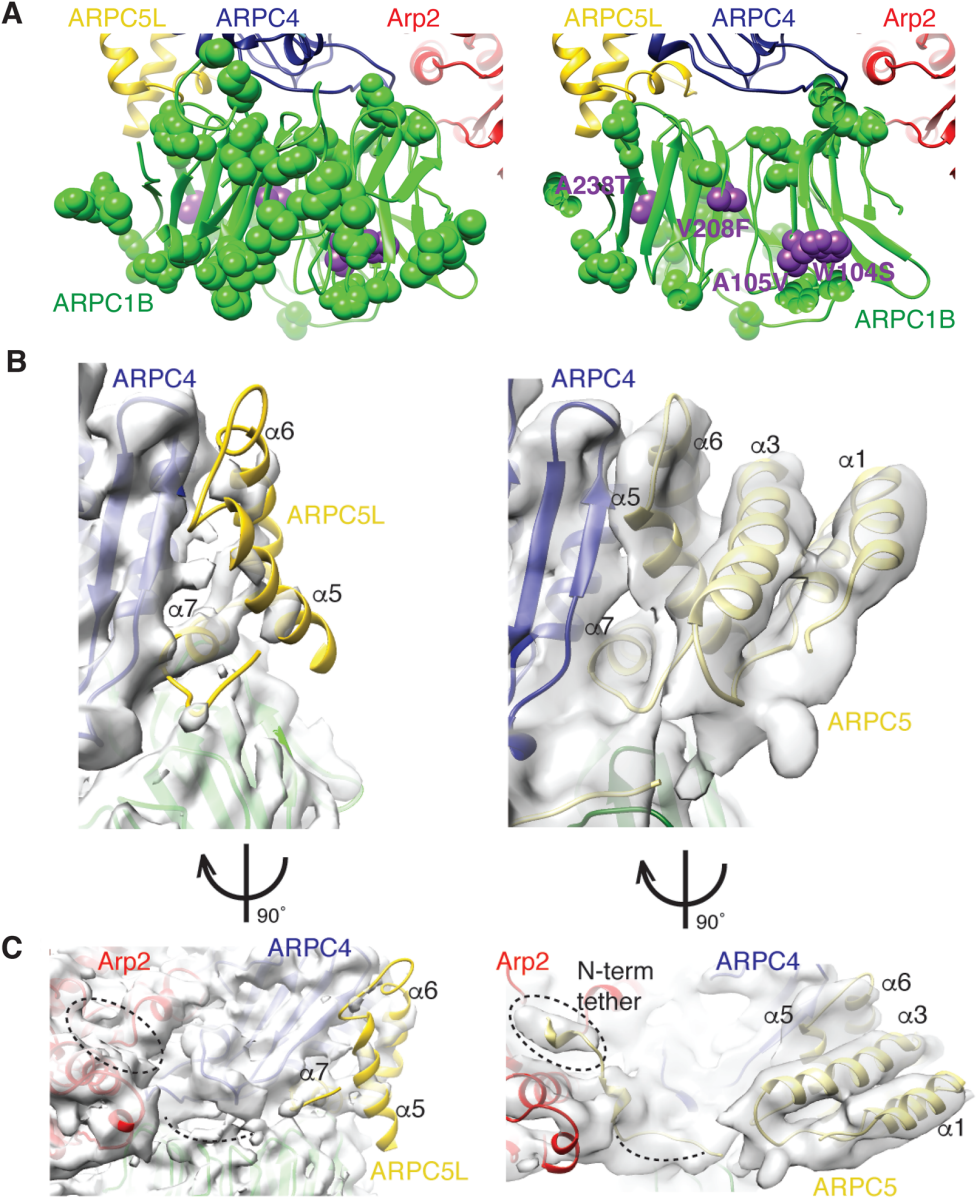
**Isoform-mediated differences in human Arp2/3 complexes.** (A) Left, location of non-conserved sequence variation between human ARPC1A and ARPC1B (green spheres) and location of disease-causing ARPC1B point mutations (purple spheres) mapped onto ARPC1B; right, cross section through ARPC1B; (B) left, density corresponding to ARPC5L, showing the incomplete density for this subunit apart from helix-α7 adjacent to ARPC4; right, density corresponding to ARPC5, showing the near complete density for this subunit (C) left, 90° rotated view compared to B, left of ARPC5L showing the incomplete density for this subunit and lack of connectivity to Arp2; right, 90° rotated view compared to B, right of ARPC5, showing the clear density for most of the subunit, including its N-terminal tether to Arp2.

To further investigate the differences between isoform-specific human Arp2/3 complexes, the location of partially- or non-conserved sequence variation between ARPC1A and ARPC1B were mapped onto the ARPC1B structure (Fig. 4A, left, green spheres; Fig. S3A). These isoform-specific-sites map primarily to the surface of the ARPC1 subunit rather than the β-propeller structural core (Fig. 4A), consistent with the overall similar structures of each isoform. This suggests that Arp2/3 activity differences arising from ARPC1 isoforms are mediated either by the interactions with ARPC4 and ARPC5 within the complex itself, and/or with other binding partners, including the mother actin filament during branch formation.

Loss-of-function mutations of ARPC1B give rise to multisystem inflammatory and immunodeficiency diseases (Brigida et al., 2018; Kahr et al., 2017; Kuijpers et al., 2017; Randzavola et al., 2019; Somech et al., 2017; Volpi et al., 2019), similar to Wiskott-Aldrich syndrome that is caused by mutations in the Arp2/3 activator WASP (Bosticardo et al., 2009). While some patients carry mutations causing premature protein termination and total loss of ARPC1B protein, four point mutations – W104S, A105V, V208F and A238T – have also been observed in patients (Brigida et al., 2018; Kahr et al., 2017; Volpi et al., 2019) (Fig. 4A, right, purple spheres). W104, A105 and V208 are located in the core of the ARPC1B subunit β-propeller fold and these mutations likely destabilises its tertiary fold, causing severely reduced protein levels in patients (Kahr et al., 2017). Conversely, A238T is more peripheral in the structure indicating that more than one disease mechanism may be in play. Although some of these patients show increased levels of ARPC1A, the activity of this isoform is insufficient to substitute for lack of functional ARPC1B, consistent with the idea that Arp2/3 complex subunit diversity has been tuned for particular physiological contexts.

The biggest difference between our two Arp2/3 cryo-EM reconstructions is the ARPC5 subunit (Fig. 4B). In the Arp2/3-C1A-C5 structure, density corresponding to the helices of ARPC5 is clearly visible (Fig. 4B, right), as is the N-terminus of the subunit that extends to contact the barbed end cleft of Arp2 (Fig. 4C, right). This was also the case in the Arp2/3-C1B-C5 complex reconstruction of (Zimmet et al., 2020). In the Arp2/3-C1B-C5L reconstruction, however, density corresponding to ARPC5L is less prominent (Fig. 4B, left). Density corresponding to the ARPC5L C-terminal helix-α7 is present and forms the major contact with the body of the Arp2/3 complex via ARPC4; at lower thresholds, density corresponding to helix-α5 and -α6 are also visible (Fig. 1B, right). However, the N-terminal half of this subunit, including connectivity to Arp2, is not visible even at low thresholds (Fig. 4C, left). In principle, this could be due to variable ARPC5L protein occupancy in the complex, but the well-defined density of helix-α7 at the interface with ARPC4 suggests this is not the case.

**Fig. 5.**
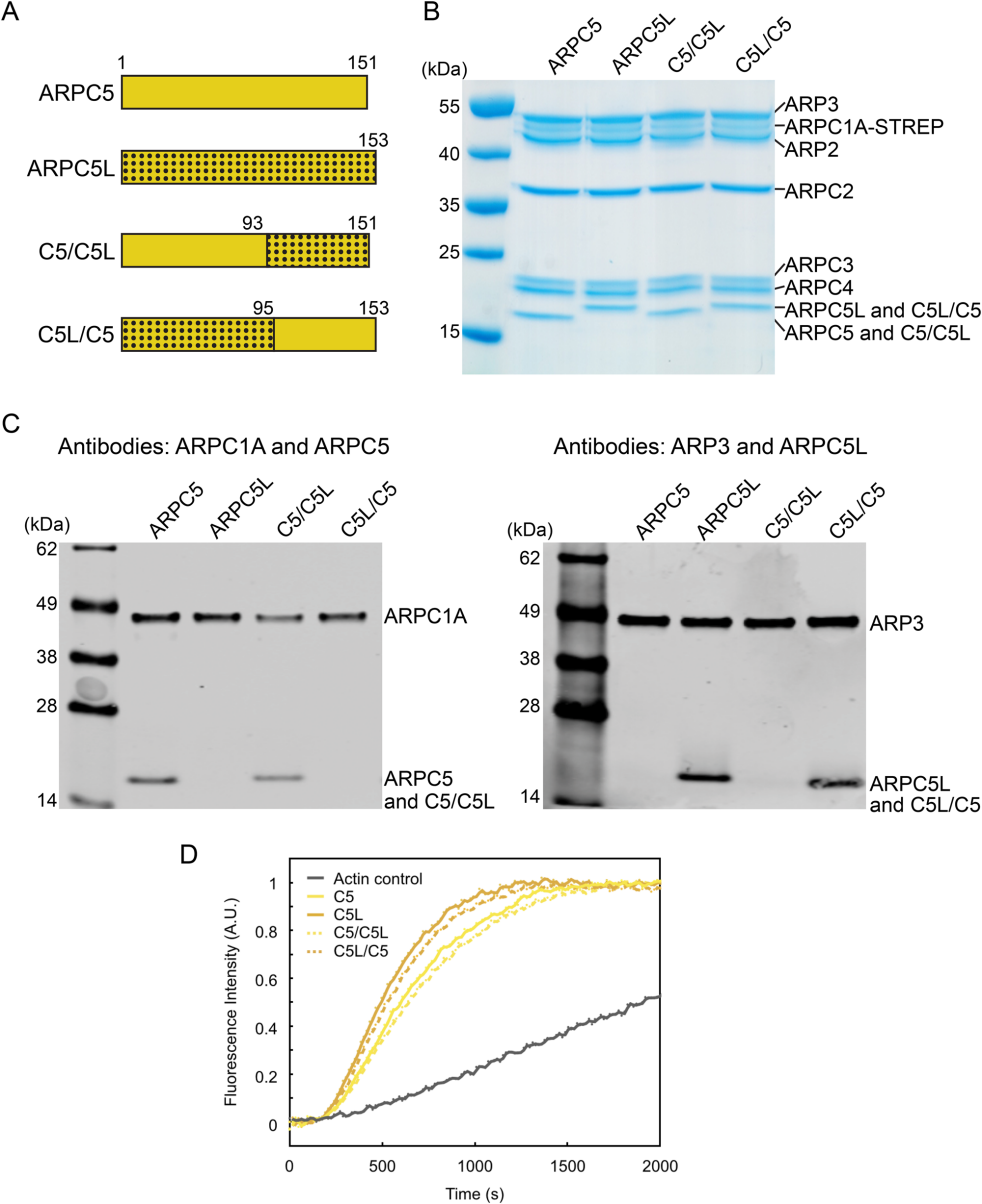
**Activity of ARPC5/C5L hybrid complexes support a role for ARPC5/C5L N-terminus in defining functional differences between Arp2/3 subunit isoforms.** (A) Schematic and nomenclature of ARPC5/C5L hybrids. (B) Coomassie-stained gel of purified recombinant Arp2/3 complexes containing ARPC1A together with ARPC5, ARPC5L or their hybrids. Gel band quantification of ARPC5, ARPC5L and the hybrids normalised to ARPC2 showed the same ratio in all cases, consistent with equivalent subunit occupancy [ARPC5/ARPC2=0.52±0.04; ARPC5L/ARPC2=0.52±0.09; C5C5L/ARPC2=0.46±0.04; C5LC5/ARPC2=0.46±0.04 (mean±s.d., *n*=3, technical replicates)]; (C) immunoblot analysis of purified recombinant Arp2/3 complexes used in this study. (D) *In vitro* polymerisation of 2 µM pyrene-actin (5% labelled), either alone (black curve) or in the presence of 5 nM VCA and 1.25 nM of Arp2/3-C1A with the indicated ARPC5 isoforms or hybrids (named as in panel A) shows differences in actin assembly according to the ARPC5/5L N-terminal region present. The curves shown here come from one representative experiment, which was repeated four times, giving similar results. The time at half-maximum, normalised to that of the ARPC5L isoform, is 1.27±0.06 for ARPC5, 1.32±0.10 for ARPC5/C5L, and 1.01±0.06 for ARPC5L/C5 (average±s.e., *n*=4, technical replicates).

The C-terminal portion of ARPC5 – visible in both isoform reconstructions – corresponds to regions of greatest sequence similarity between the isoforms and between organisms, whereas the N-terminal region is more divergent (Fig. S3B). Our structures suggest the intriguing possibility that the higher activity of Arp2/3-C1B-C5L complexes (Abella et al., 2016) may be due to the intrinsically more flexible attachment of ARPC5L N-terminus to the complex. To test this idea, we engineered ARPC5/C5L protein hybrids, in which their N-terminal regions were swapped (Fig. 5A–C; Fig. S3B), and tested their actin nucleation activity in the context of the lowest activity Arp2/3-C1A complex. Consistent with the prediction arising from our structures, the hybrid complex containing the N-terminal region of ARPC5L was more active than that containing the equivalent region of ARPC5 (Fig. 5D).

We therefore suggest that while the C-terminus of ARPC5 is closely associated with the body of Arp2/3, the role of its N-terminus is to contribute to regulation of complex activation: when contacting Arp2, the complex is held in an inactive conformation, while priming of the complex involves release of these contacts and conformational restraints between the subunits. With its looser, less structured N-terminal connectivity, human ARPC5L-containing complexes are intrinsically more able to undergo activator-induced conformational changes, and may also adopt alternative conformations in the activated complex (Dalhaimer and Pollard, 2010). All currently available X-ray crystal structures of mammalian Arp2/3 complexes contain the ARPC5 isoform and most of these structures fully visualise ARPC5; however, some also exhibit ARPC5 N-terminal conformational variability. Together with lower resolution negative stain EM analysis of *Saccharomyces cerevisiae* Arp2/3 complexes (Martin et al., 2005) and NPF-bound *Schizosaccharomyces pombe* Arp2/3 complex (Espinoza-Sanchez et al., 2018), these observations support the wider functional relevance of the dynamics of the ARPC5 N-terminus in Arp2/3 activation revealed in our cryo-EM reconstructions.

The overall conformations of our ATP-bound Arp2/3 cryo-EM structures are similar to previously determined X-ray crystallography structures of natively purified bovine Arp2/3 complexes (Nolen et al., 2004; Nolen and Pollard, 2007). Earlier lower resolution negative stain EM studies of Arp2/3 complex have described a range of conformational changes in the Arp2/3 complex in response to ATP binding that likely reflect the intrinsic flexibility of portions of the unactivated complex, especially within Arp2 (Rodal et al., 2005; Sokolova et al., 2017). Thus, rather than inducing major conformational changes in the complex, available structures of Arp2/3 together with biophysical measurements point to the role of ATP binding in potentiating subsequent conformational changes induced by other activating factors, including nucleation promoting factors and binding to the mother filament (Goley and Welch, 2006; Kiselar et al., 2007; Luan et al., 2018; Martin et al., 2005; Zimmet et al., 2020). Our results set the stage for cryo-EM structure determination of Arp2/3 complexes bound by nucleation promoting factors, promising insights concerning steps of Arp2/3 activation – including isoform-dependent modulation of complex activation – in the future.

## MATERIALS AND METHODS

### Cryo-EM grid preparation and data collection

Arp2/3-C1B-C5L and Arp2/3-C1A-C5 complexes were prepared using the multibac expression system consisting of three pFL vectors (pFL–ARPC2–Arp3; pFL–ARPC4–ARPC1A or C1B–STREP; and pFL–ARPC3–ARP2–ARPC5 or C5L) as described previously (Abella et al., 2016). They were diluted to 0.2–0.3 mg/ml in MOPS buffer (20 mM MOPS pH 7.0, 100 mM KCl, 2 mM MgCl_2_, 5 mM EGTA, 1 mM EDTA, 0.5 mM DTT, 0.2 mM ATP) and 4 µl were applied to glow-discharged 1.2/1.3 holey gold grids (Quantifoil) before plunge freezing in liquid ethane using a Vitrobot (FEI/Thermo Fisher Scientific) operating at room temperature and 100% humidity. Data for the Arp2/3-C1B-C5L complexes were collected at the UK National Electron Bio-imaging Centre (eBIC) at the Diamond Synchrotron using EPU on a Titan Krios microscope (FEI/Thermo Fisher Scientific) operating at 300 kV, and recorded on a K2 direct electron detector equipped with a Quantum energy filter (Gatan), with a pixel size of 1.06 Å/px. Two movies per hole were collected using a total dose of 60 e^−^/Å^2^ each over 20 frames with 8 s exposure, and with a defocus range of −1.5 – −3.5 µm. Data for the Arp2/3-C1A-C5 complexes were collected manually on a Polara microscope (FEI/Thermo Fisher Scientific) operating at 300 kV, and recorded on a K2 direct electron detector equipped with a Quantum energy filter (Gatan), with a pixel size of 1.09 Å/px. A total dose of 59 e^−^/Å^2^ per movie over 33 frames with 10 s exposure, and with a defocus range of −1.5 – −3.5 µm.

For movie processing, all frames were aligned with using MotionCor2 (Zheng et al., 2017) and CTF was estimated with ctffind4 (Rohou and Grigorieff, 2015). Each dataset was processed independently using the RELION beta-version 2.0 (Kimanius et al., 2016). For the Arp2/3-C1B-C5L dataset, 1,547,329 particles were picked automatically from 3039 micrographs in RELION v1.4 and v2.0 (Kimanius et al., 2016; Scheres, 2012), with a box-size of 240×240 pixels. After iterative cleaning of the particles using 2D classification and particle sorting, 384,330 particles remained. These were subjected to a first 3D refinement using a crystal structure of the bovine ARP2/3 complex (PDB: 3DXM; Nolen et al., 2009) converted to EM density in EMAN (Ludtke et al., 1999) and filtered to 20 Å as an initial reference. The resulting initial reconstruction was used as a reference for several further rounds of 2D and 3D classification in RELION. The final 3D-reconstruction containing 101,606 particles was calculated from dose-weighted data and was automatically B-factor sharpened in RELION using with a B-factor of −151 (Scheres, 2012). The final overall resolution of the masked reconstruction was 4.2 Å (0.143 gold-standard-FSC). For the Arp2/3-C1A-C5 dataset, a similar procedure was followed: 697,272 particles were picked from 1221 micrographs with a box-size of 256×256 pixels. After iterative cleaning of the particles using 2D classification and particle sorting, 405,152 particles remained. Because of the highly preferred orientation of particles in this dataset, particles with over-represented orientations were removed by 2D and 3D classification. The final 3D-reconstruction containing 130,973 particles was calculated from dose-weighted data and was automatically B-factor sharpened in RELION using with a B-factor of −179 (Scheres, 2012). The final overall resolution of the masked reconstruction was 4.5 Å.

### Model building

An initial model of Arp2/3-C1B-C5L was created using coordinates from PDB file 1K8K, and was rigid-body docked to the cryo-EM map using Chimera (Pettersen et al., 2004). Additional elements including the nucleotides from PDB file 4XF2 were added, before the bovine sequences from these structures were altered to human. Secondary structure-based rigid bodies were described with Ribfind (Pandurangan and Topf, 2012), before a combination of conjugate gradient energy minimisation and molecular dynamics fitting of the model into the map was undertaken with Flex-EM (Topf et al., 2008), part of the CCP-EM suite (Burnley et al., 2017). The final model was processed using real space refinement in Phenix (Adams et al., 2010), for which a refinement resolution cut-off of 5 Å was used. Molprobity (Chen et al., 2010) was utilised to validate the geometry of the resultant model, which was then improved by manual inspection and local refinement of poor areas in Coot (Emsley et al., 2010), with Ramachandran and secondary structure restraints utilised. Because the Arp2/3-C1A-C5 reconstruction showed substantial non-isotropic resolution, a separate model for this structure was not calculated. Instead, the refined Arp2/3-C1B-C5L model was rigidly docked into the Arp2/3-C1A-C5 density, the ARPC5 model was replaced with that from 1K8K and its docking was locally refined. A full validation report for the atomic model was generated in phenix v1.17.1-3660 (Table 1).

### Production of ARPC5/C5L hybrid complexes

ARPC5/C5L hybrid-containing complexes were designed by replacing residues 1-95 of C5L with 1-93 of C5, while for ARPC5L/C5 residues 96-153 of C5L were replaced with 94-151 of C5 (Fig. 5A). DNA corresponding to the hybrids were obtained from GeneArt Gene synthesis (Thermo Fisher Scientific), which were cloned into pFL vector to obtain pFL-ARPC5/C5L using BamHI/NotI sites and pFL-ARPC5L/C5 using XhoI/XmaI sites, respectively. pFL-ARPC5/C5L was digested using BstZ17I/AvrII and the resulting insert was ligated into pFL-ARPC3-ACTR2 that was linearised using BstZ17I/SpeI, to create pFL-ARPC3-ACTR2-ARPC5/C5L. pFL-ARPC5L/C5 was digested using BstZ17I/PmeI and the insert was ligated into pFL-ARPC3-ACTR2 that was linearised with BstZ17I, to generate pFL-ARPC3-ACTR2-ARPC5L/C5. The expression in Sf21 insect cells and protein purification of ARP2/3 complexes containing the hybrids was performed as previously described (Fig. 5B) (Abella et al., 2016). Gel band quantification was performed using Fiji (Schindelin et al., 2012). To validate expression of the hybrids, Near-infrared Western immunoblot using Odyssey CLx detection system (Li-COR) was performed on the purified complexes with the following antibodies: Arp3 (Sigma-Aldrich A5979, mouse), ARPC5L (Abcam ab169763, rabbit), ARPC1A (Sigma-Aldrich HPA004334, rabbit), and ARPC5 (Santa Cruz Biotechnology sc-166760, mouse) (Fig. 5C).

### Actin nucleation assays

Recombinant GST-tagged VCA domain of human N-WASP (391-505) was expressed in E.Coli Rosetta 2 (DE3) and purified by affinity chromatography over a Sepharose 4B GSH affinity column (GE Healthcare) followed by gel filtration over a Hiload Superdex 200 column (GE Healthcare). Skeletal muscle actin was purified from rabbit muscle acetone powder following the protocol described in Wioland et al. (2017). Pyrenyl-actin was made by labelling actin with N(1-pyrene)-iodoacetamide (Thermo Fisher Scientific).

Actin assembly was detected by the change in pyrenyl-actin fluorescence using a Safas Xenius spectrofluorimeter (Safas) at room temperature. 1 μl of 0.2 μM Arp2/3, 1 μl of 0.8 μM VCA and 8 μl of 20xKME (4 mM EGTA, 20 mM MgCl_2_, 1 M KCl) were mixed with 110 µl of G-buffer (10 mM Tris HCl pH 7.0, 0.2 mM ATP, 0.1 mM CaCl_2_, 1 mM DTT). 40 μl of 8 μΜ Mg-ATP-G-actin (5% pyrene labelled) was added to this protein solution and mixed rapidly. The fluorescence signal was recorded immediately, and until the curves reached the steady-state plateau. The fluorescence intensity was normalised using I(t) = (I_obs_(t)-I_min_)/(I_max_-I_min_) where I_min_, the average of the ten lowest data points, refers to the signal intensity before actin started to polymerise and I_max_, the average of the ten highest data points, refers to the signal intensity at steady-state. The experiments were repeated four times, giving similar results. The different data sets were not combined.

## Supplementary Material

Supplementary information

## References

[BIO054304C1] Abella J. V. G., Galloni C., Pernier J., Barry D. J., Kjær S., Carlier M. F. and Way M. (2016). Isoform diversity in the Arp2/3 complex determines actin filament dynamics. *Nat. Cell Biol.* 18, 76-86. 10.1038/ncb3286 26655834

[BIO054304C2] Adams P. D., Afonine P. V., Bunkóczi G., Chen V. B., Davis I. W., Echols N., Headd J. J., Hung L.-W., Kapral G. J., Grosse-Kunstleve R. W. et al. (2010). Phenix: a comprehensive python-based system for macromolecular structure solution. *Acta Crystallogr. D Biol. Crystallogr.* 66, 213-221. 10.1107/S0907444909052925 20124702 PMC2815670

[BIO054304C3] Bosticardo M., Marangoni F., Aiuti A., Villa A. and Grazia Roncarolo M. (2009). Recent advances in understanding the pathophysiology of Wiskott-Aldrich syndrome. *Blood* 113, 6288-6295. 10.1182/blood-2008-12-115253 19351959

[BIO054304C4] Brigida I., Zoccolillo M., Cicalese M. P., Pfajfer L., Barzaghi F., Scala S., Oleaga-Quintas C., Álvarez-Álvarez J. A., Sereni L., Giannelli S. et al. (2018). T-cell defects in patients with ARPC1B germline mutations account for combined immunodeficiency. *Blood* 132, 2362-2374. 10.1182/blood-2018-07-863431 30254128 PMC6265646

[BIO054304C5] Burnley T., Palmer C. M. and Winn M. (2017). Recent developments in the CCP-EM software suite. *Acta Crystallogr. D Struct. Biol.* 73, 469-477. 10.1107/S2059798317007859 28580908 PMC5458488

[BIO054304C6] Campellone K. G. and Welch M. D. (2010). A nucleator arms race: cellular control of actin assembly. *Nat. Rev. Mol. Cell Biol.* 11, 237-251. 10.1038/nrm2867 20237478 PMC2929822

[BIO054304C7] Caridi C. P., D'agostino C., Ryu T., Zapotoczny G., Delabaere L., Li X., Khodaverdian V. Y., Amaral N., Lin E., Rau A. R. et al. (2018). Nuclear F-actin and myosins drive relocalization of heterochromatic breaks. *Nature* 559, 54-60. 10.1038/s41586-018-0242-8 29925946 PMC6051730

[BIO054304C8] Chen V. B., Arendall W. B. III, Headd J. J., Keedy D. A., Immormino R. M., Kapral G. J., Murray L. W., Richardson J. S. and Richardson D. C. (2010). MolProbity: all-atom structure validation for macromolecular crystallography. *Acta Crystallogr. D Biol. Crystallogr.* 66, 12-21. 10.1107/S0907444909042073 20057044 PMC2803126

[BIO054304C9] Dalhaimer P. and Pollard T. D. (2010). Molecular dynamics simulations of Arp2/3 complex activation. *Biophys. J.* 99, 2568-2576. 10.1016/j.bpj.2010.08.027 20959098 PMC2955496

[BIO054304C10] Emsley P., Lohkamp B., Scott W. G. and Cowtan K. (2010). Features and development of Coot. *Acta Crystallogr. D Biol. Crystallogr.* 66, 486-501. 10.1107/S0907444910007493 20383002 PMC2852313

[BIO054304C11] Espinoza-Sanchez S., Metskas L. A., Chou S. Z., Rhoades E. and Pollard T. D. (2018). Conformational changes in Arp2/3 complex induced by ATP, WASp-VCA, and actin filaments. *Proc. Natl. Acad. Sci. USA* 115, E8642-E8651. 10.1073/pnas.1717594115 30150414 PMC6140485

[BIO054304C12] Goley E. D. and Welch M. D. (2006). The ARP2/3 complex: an actin nucleator comes of age. *Nat. Rev. Mol. Cell Biol.* 7, 713-726. 10.1038/nrm2026 16990851

[BIO054304C13] Ingerman E., Hsiao J. Y. and Mullins R. D. (2013). Arp2/3 complex ATP hydrolysis promotes lamellipodial actin network disassembly but is dispensable for assembly. *J. Cell Biol.* 200, 619-633. 10.1083/jcb.201211069 23439681 PMC3587832

[BIO054304C14] Kahr W. H. A., Pluthero F. G., Elkadri A., Warner N., Drobac M., Chen C. H., Lo R. W., Li L., Li R., Li Q. et al. (2017). Loss of the Arp2/3 complex component ARPC1B causes platelet abnormalities and predisposes to inflammatory disease. *Nat. Commun.* 8, 14816 10.1038/ncomms14816 28368018 PMC5382316

[BIO054304C15] Kimanius D., Forsberg B. O., Scheres S. H. and Lindahl E. (2016). Accelerated cryo-EM structure determination with parallelisation using GPUs in RELION-2. *eLife* 5, e18722 10.7554/elife.18722 27845625 PMC5310839

[BIO054304C16] Kiselar J. G., Mahaffy R., Pollard T. D., Almo S. C. and Chance M. R. (2007). Visualizing Arp2/3 complex activation mediated by binding of ATP and WASp using structural mass spectrometry. *Proc. Natl. Acad. Sci. USA* 104, 1552-1557. 10.1073/pnas.0605380104 17251352 PMC1785275

[BIO054304C17] Krause M. and Gautreau A. (2014). Steering cell migration: lamellipodium dynamics and the regulation of directional persistence. *Nat. Rev. Mol. Cell Biol.* 15, 577-590. 10.1038/nrm3861 25145849

[BIO054304C18] Kuijpers T. W., Tool A. T. J., Van Der Bijl I., De Boer M., Van Houdt M., De Cuyper I. M., Roos D., Van Alphen F., Van Leeuwen K., Cambridge E. L. et al. (2017). Combined immunodeficiency with severe inflammation and allergy caused by ARPC1B deficiency. *J. Allergy Clin. Immunol.* 140, 273-277.e10. 10.1016/j.jaci.2016.09.061 27965109

[BIO054304C19] Le Clainche C., Didry D., Carlier M.-F. and Pantaloni D. (2001). Activation of Arp2/3 complex by Wiskott-Aldrich Syndrome protein is linked to enhanced binding of ATP to Arp2. *J. Biol. Chem.* 276, 46689-46692. 10.1074/jbc.C100476200 11598103

[BIO054304C20] Luan Q. and Nolen B. J. (2013). Structural basis for regulation of Arp2/3 complex by GMF. *Nat. Struct. Mol. Biol.* 20, 1062-1068. 10.1038/nsmb.2628 23893131 PMC3766443

[BIO054304C21] Luan Q., Liu S.-L., Helgeson L. A. and Nolen B. J. (2018). Structure of the nucleation-promoting factor SPIN 90 bound to the actin filament nucleator Arp2/3 complex. *EMBO J.* 37, e100005 10.15252/embj.2018100005 30322896 PMC6236337

[BIO054304C22] Ludtke S. J., Baldwin P. R. and Chiu W. (1999). EMAN: semiautomated software for high-resolution single-particle reconstructions. *J. Struct. Biol.* 128, 82-97. 10.1006/jsbi.1999.4174 10600563

[BIO054304C23] Martin A. C., Xu X.-P., Rouiller I., Kaksonen M., Sun Y., Belmont L., Volkmann N., Hanein D., Welch M. and Drubin D. G. (2005). Effects of Arp2 and Arp3 nucleotide-binding pocket mutations on Arp2/3 complex function. *J. Cell Biol.* 168, 315-328. 10.1083/jcb.200408177 15657399 PMC2171590

[BIO054304C24] Martin A. C., Welch M. D. and Drubin D. G. (2006). Arp2/3 ATP hydrolysis-catalysed branch dissociation is critical for endocytic force generation. *Nat. Cell Biol.* 8, 826-833. 10.1038/ncb1443 16862144

[BIO054304C25] Molinie N. and Gautreau A. (2018). The Arp2/3 regulatory system and its deregulation in cancer. *Physiol. Rev.* 98, 215-238. 10.1152/physrev.00006.2017 29212790

[BIO054304C26] Nolen B. J. and Pollard T. D. (2007). Insights into the influence of nucleotides on actin family proteins from seven structures of Arp2/3 complex. *Mol. Cell* 26, 449-457. 10.1016/j.molcel.2007.04.017 17499050 PMC1997283

[BIO054304C27] Nolen B. J. and Pollard T. D. (2008). Structure and biochemical properties of fission yeast Arp2/3 complex lacking the Arp2 subunit. *J. Biol. Chem.* 283, 26490-26498. 10.1074/jbc.M802607200 18640983 PMC2546537

[BIO054304C28] Nolen B. J., Littlefield R. S. and Pollard T. D. (2004). Crystal structures of actin-related protein 2/3 complex with bound ATP or ADP. *Proc. Natl. Acad. Sci. USA* 101, 15627-15632. 10.1073/pnas.0407149101 15505213 PMC524860

[BIO054304C29] Nolen B. J., Tomasevic N., Russell A., Pierce D. W., Jia Z., Mccormick C. D., Hartman J., Sakowicz R. and Pollard T. D. (2009). Characterization of two classes of small molecule inhibitors of Arp2/3 complex. *Nature* 460, 1031-1034. 10.1038/nature08231 19648907 PMC2780427

[BIO054304C30] Pandurangan A. P. and Topf M. (2012). RIBFIND: a web server for identifying rigid bodies in protein structures and to aid flexible fitting into cryo EM maps. *Bioinformatics* 28, 2391-2393. 10.1093/bioinformatics/bts446 22796953

[BIO054304C31] Pettersen E. F., Goddard T. D., Huang C. C., Couch G. S., Greenblatt D. M., Meng E. C. and Ferrin T. E. (2004). UCSF Chimera--a visualization system for exploratory research and analysis. *J. Comput. Chem.* 25, 1605-1612. 10.1002/jcc.20084 15264254

[BIO054304C32] Pizarro-Cerdá J., Chorev D. S., Geiger B. and Cossart P. (2017). The diverse family of Arp2/3 Complexes. *Trends Cell Biol.* 27, 93-100. 10.1016/j.tcb.2016.08.001 27595492 PMC7098815

[BIO054304C33] Randzavola L. O., Strege K., Juzans M., Asano Y., Stinchcombe J. C., Gawden-Bone C. M., Seaman M. N. J., Kuijpers T. W. and Griffiths G. M. (2019). Loss of ARPC1B impairs cytotoxic T lymphocyte maintenance and cytolytic activity. *J. Clin. Invest.* 129, 5600-5614. 10.1172/JCI129388 31710310 PMC6877333

[BIO054304C34] Robinson R. C., Turbedsky K., Kaiser D. A., Marchand J. B., Higgs H. N., Choe S. and Pollard T. D. (2001). Crystal structure of Arp2/3 complex. *Science* 294, 1679-1684. 10.1126/science.1066333 11721045

[BIO054304C35] Rodal A. A., Sokolova O., Robins D. B., Daugherty K. M., Hippenmeyer S., Riezman H., Grigorieff N. and Goode B. L. (2005). Conformational changes in the Arp2/3 complex leading to actin nucleation. *Nat. Struct. Mol. Biol.* 12, 26-31. 10.1038/nsmb870 15592479

[BIO054304C36] Rodnick-Smith M., Liu S.-L., Balzer C. J., Luan Q. and Nolen B. J. (2016). Identification of an ATP-controlled allosteric switch that controls actin filament nucleation by Arp2/3 complex. *Nat. Commun.* 7, 12226 10.1038/ncomms12226 27417392 PMC4947185

[BIO054304C37] Rohou A. and Grigorieff N. (2015). CTFFIND4: fast and accurate defocus estimation from electron micrographs. *J. Struct. Biol.* 192, 216-221. 10.1016/j.jsb.2015.08.008 26278980 PMC6760662

[BIO054304C38] Roman W., Martins J. P., Carvalho F. A., Voituriez R., Abella J. V. G., Santos N. C., Cadot B., Way M. and Gomes E. R. (2017). Myofibril contraction and crosslinking drive nuclear movement to the periphery of skeletal muscle. *Nat. Cell Biol.* 19, 1189-1201. 10.1038/ncb3605 28892082 PMC5675053

[BIO054304C39] Scheres S. H. W. (2012). RELION: implementation of a Bayesian approach to cryo-EM structure determination. *J. Struct. Biol.* 180, 519-530. 10.1016/j.jsb.2012.09.006 23000701 PMC3690530

[BIO054304C40] Schindelin J., Arganda-Carreras I., Frise E., Kaynig V., Longair M., Pietzsch T., Preibisch S., Rueden C., Saalfeld S., Schmid B. et al. (2012). Fiji: an open-source platform for biological-image analysis. *Nat. Methods* 9, 676-682. 10.1038/nmeth.2019 22743772 PMC3855844

[BIO054304C41] Schrank B. R., Aparicio T., Li Y., Chang W., Chait B. T., Gundersen G. G., Gottesman M. E. and Gautier J. (2018). Nuclear ARP2/3 drives DNA break clustering for homology-directed repair. *Nature* 559, 61-66. 10.1038/s41586-018-0237-5 29925947 PMC6145447

[BIO054304C42] Sokolova O. S., Chemeris A., Guo S., Alioto S. L., Gandhi M., Padrick S., Pechnikova E., David V., Gautreau A. and Goode B. L. (2017). Structural basis of Arp2/3 complex inhibition by GMF, coronin, and arpin. *J. Mol. Biol.* 429, 237-248. 10.1016/j.jmb.2016.11.030 27939292 PMC5350076

[BIO054304C43] Somech R., Lev A., Lee Y. N., Simon A. J., Barel O., Schiby G., Avivi C., Barshack I., Rhodes M., Yin J. et al. (2017). Disruption of thrombocyte and T lymphocyte development by a mutation in ARPC1B. *J. Immunol.* 199, 4036-4045. 10.4049/jimmunol.1700460 29127144 PMC5726601

[BIO054304C44] Topf M., Lasker K., Webb B., Wolfson H., Chiu W. and Sali A. (2008). Protein structure fitting and refinement guided by cryo-EM density. *Structure* 16, 295-307. 10.1016/j.str.2007.11.016 18275820 PMC2409374

[BIO054304C45] Volpi S., Cicalese M. P., Tuijnenburg P., Tool A. T. J., Cuadrado E., Abu-Halaweh M., Ahanchian H., Alzyoud R., Akdemir Z. C., Barzaghi F. et al. (2019). A combined immunodeficiency with severe infections, inflammation, and allergy caused by ARPC1B deficiency. *J. Allergy Clin. Immunol.* 143, 2296-2299. 10.1016/j.jaci.2019.02.003 30771411 PMC6677392

[BIO054304C46] Wioland H., Guichard B., Senju Y., Myram S., Lappalainen P., Jégou A. and Romet-Lemonne G. (2017). ADF/cofilin accelerates actin dynamics by severing filaments and promoting their depolymerization at both ends. *Curr. Biol.* 27, 1956-1967.e7. 10.1016/j.cub.2017.05.048 28625781 PMC5505867

[BIO054304C47] Zheng S. Q., Palovcak E., Armache J.-P., Verba K. A., Cheng Y. and Agard D. A. (2017). MotionCor2: anisotropic correction of beam-induced motion for improved cryo-electron microscopy. *Nat. Methods* 14, 331-332. 10.1038/nmeth.4193 28250466 PMC5494038

[BIO054304C48] Zimmet A., Van Eeuwen T., Boczkowska M., Rebowski G., Murakami K. and Dominguez R. (2020). Cryo-EM structure of NPF-bound human Arp2/3 complex and activation mechanism. *Sci. Adv.* 6, eaaz7651 10.1126/sciadv.aaz7651 32917641 PMC7274804

