# Supplementary material for: Cryo-EM of human Arp2/3 complexes provides structural insights into actin nucleation modulation by ARPC5 isoforms
Source: Biol Open. 2020 Jul 31;9(7):bio054304. doi: 10.1242/bio.054304 (PMC7406314; doi:10.1242/bio.054304)
Supplement: Supplementary information [file biolopen-9-054304-s1.pdf]

Cryo-EM of human Arp2/3 complexes provides structural insights into actin nucleation modulation by ARPC5 isoforms

Ottilie von Loeffelholz, Andrew Purkiss, Luyan Cao, Svend Kjaer, Naoko Kogata, Guillaume Romet-Lemonne, Michael Way, & Carolyn A. Moores

SUPPLEMENTARY FIGURES

Biology Open • Supplementary information

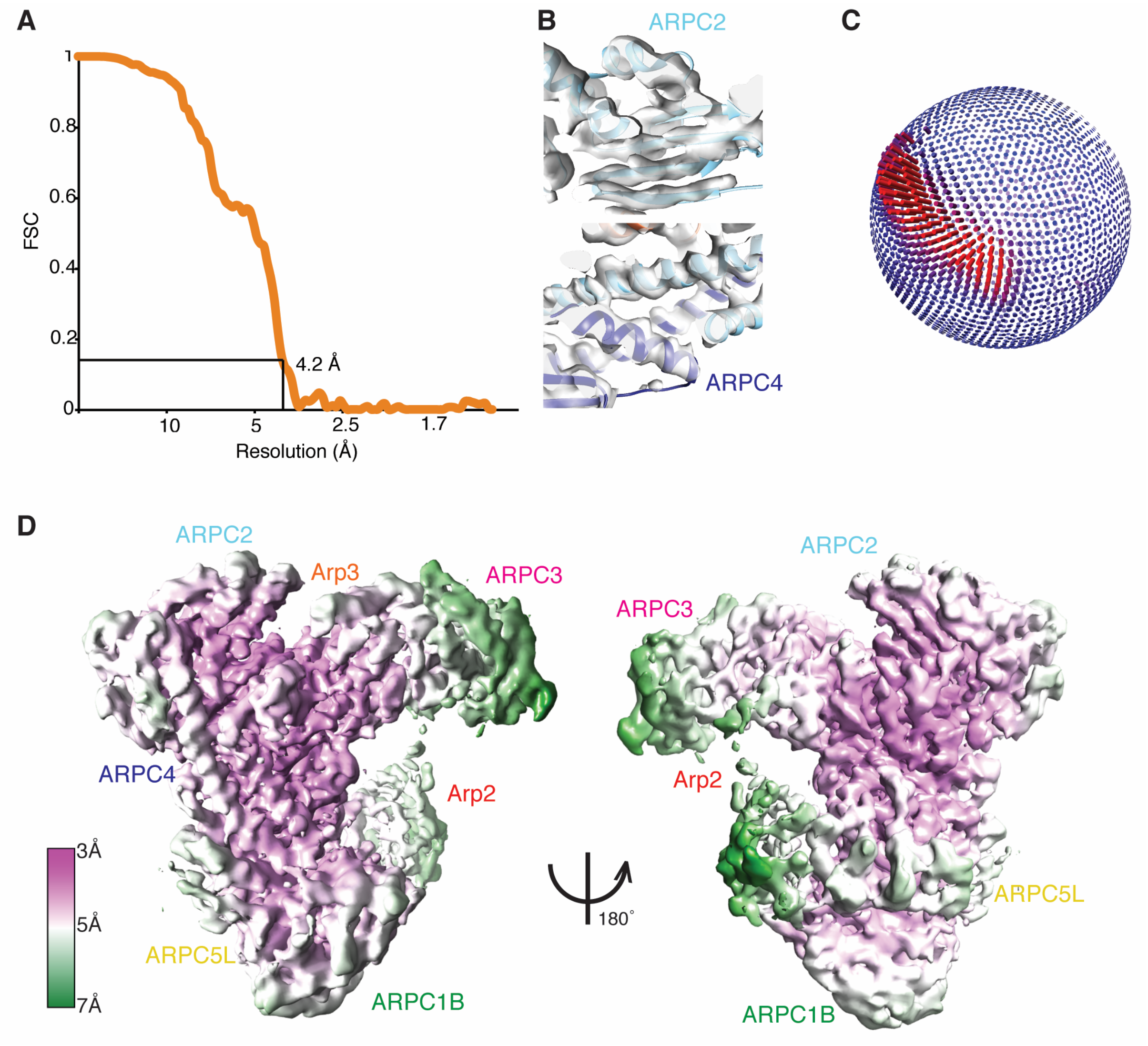

Figure S1. Evaluation of the resolution of the Arp2/3-C1B-C5L reconstruction.

(A) FSC curve of the Arp2/3-C1B-C5L reconstruction, showing the 0.143 criteria resolution cut-off = 4.2 Å;
(B) Example sections of the Arp2/3-C1B-C5L cryo-EM density showing a β-sheet region of ARPC2 (top) and α-helical regions of ARPC2 and ARPC4 (bottom) illustrating the quality of the best regions of the reconstruction;
(C) Orientation distribution of particles used for the final 3D reconstruction; the most common view corresponds to that depicted in Figure 1A;
(D) Local resolution depiction of the Arp2/3-C1B-C5L reconstruction calculated using RELION, showing views equivalent to Figure 1A (left) and Figure 1B (right).

Biology Open • Supplementary information

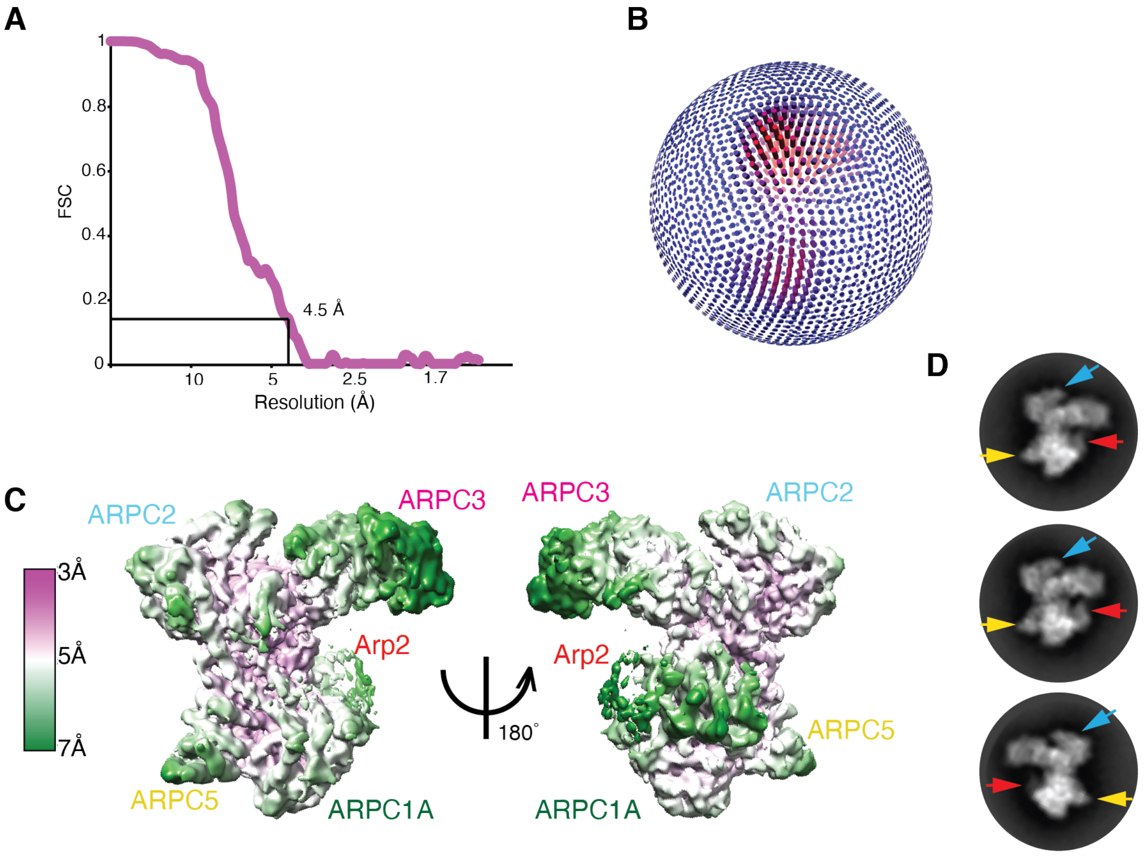

Figure S2. Evaluation of the resolution of the Arp2/3-ARPC1A-ARPC5 reconstruction.

(A) FSC curve of the Arp2/3-C1A-C5 reconstruction, showing the 0.143 criteria resolution cut-off = 4.5Å;
(B) Orientation distribution of particles used for the final 3D reconstruction; the most common view corresponds to that depicted in Figure 3A;
(C) Local resolution depiction of the Arp2/3-C1A-C5 reconstruction calculated using RELION, showing views equivalent to panel A (left) and panel B (right).
(D) 2D class averages of Arp2/3-C1A-C5 showing views corresponding to Figure 3A (upper 2 classes) and Figure 3B (bottom panel) illustrating the variable density corresponding to subdomain 2 of Arp2 (red arrows) but the consistent density corresponding to ARPC5 (yellow arrows); this contrasts to that seen in the Arp2/3-C1B-C5L complex. ARPC2 (blue arrow) is also indicated for reference.

Biology Open • Supplementary information

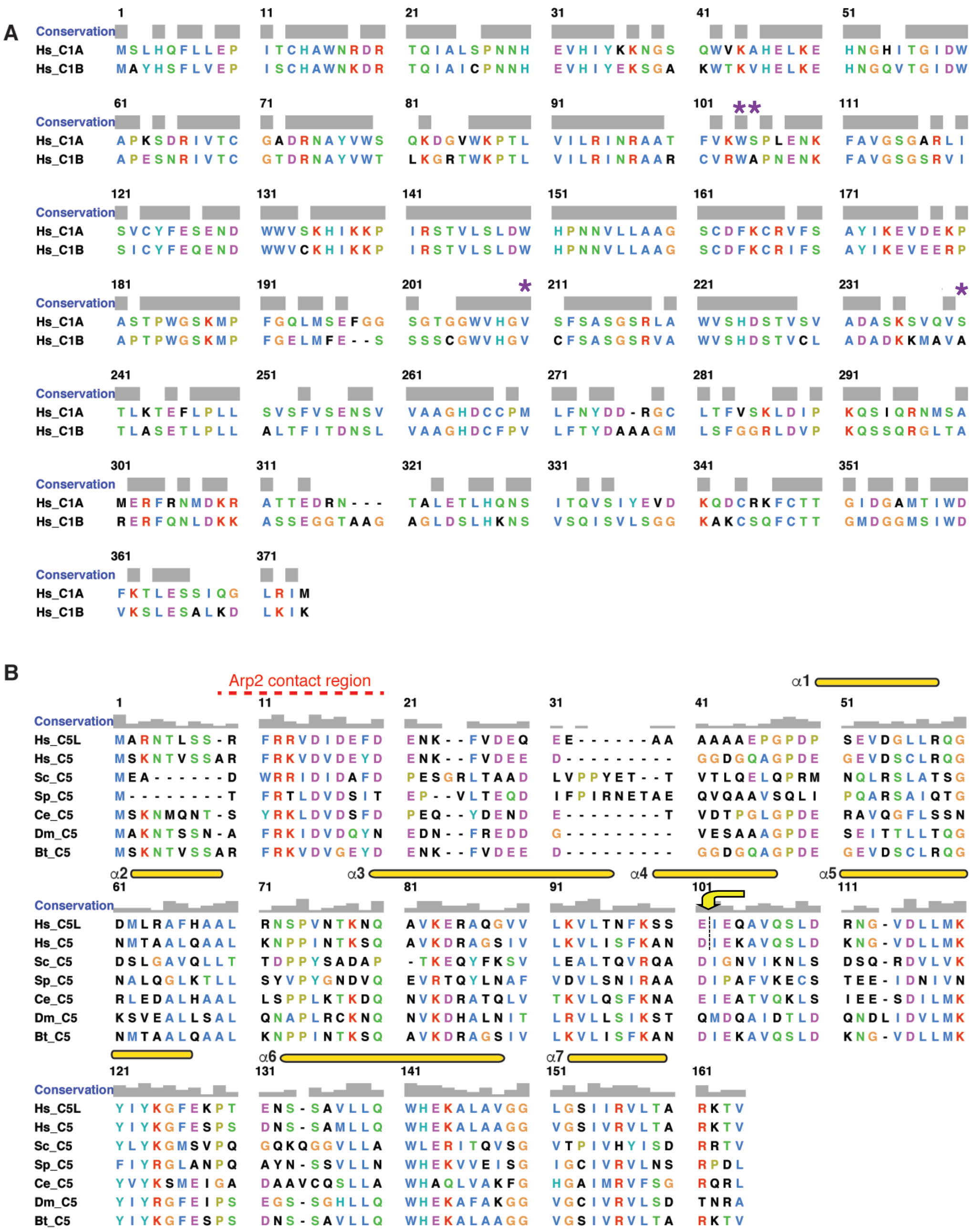

Figure S3. Sequence alignment of ARPC1 and ARPC5.
(A) Sequence alignment of human ARPC1 isoforms. Sequences were aligned using T-Coffee (Notredame et al., 2000) and the alignment was prepared using Chimera (Pettersen et al., 2004) with the Clustal X colour scheme. The position of immunodeficiency syndrome-associated point mutations in ARCP1B – W104S, A105V, V208F and A238T – are indicated with purple asterisks (Kahr et al., 2017).
(B) Sequence alignment of ARPC5 including the two human isoforms (Hs_C5L, Hs_C5), and C5 sequences from *S. cerevisiae* (Sc_S5), *S. pombe* (Sp_C5), *C. elegans* (Ce_C5), *D. melanogaster* (Dm_C5) and *B. taurus* (Bt_C5). As, above, sequences were aligned using T-Coffee, the alignment was prepared using Chimera with the Clustal X colour scheme, and the main secondary structural elements are annotated above the alignment according to (Robinson et al., 2001). The yellow arrow/dotted line indicates the splicepoint in the C5/C5L hybrids.

Biology Open • Supplementary information

References

Kahr, W.H., Pluthero, F.G., Elkadri, A., Warner, N., Drobac, M., Chen, C.H., Lo, R.W., Li, L., Li, R., Li, Q., *et al.* (2017). Loss of the Arp2/3 complex component ARPC1B causes platelet abnormalities and predisposes to inflammatory disease. Nat Commun *8*, 14816.
Notredame, C., Higgins, D.G., and Heringa, J. (2000). T-Coffee: A novel method for fast and accurate multiple sequence alignment. J Mol Biol *302*, 205-217.
Pettersen, E.F., Goddard, T.D., Huang, C.C., Couch, G.S., Greenblatt, D.M., Meng, E.C., and Ferrin, T.E. (2004). UCSF Chimera--a visualization system for exploratory research and analysis. J Comput Chem *25*, 1605-1612.
Robinson, R.C., Turbedsky, K., Kaiser, D.A., Marchand, J.B., Higgs, H.N., Choe, S., and Pollard, T.D. (2001). Crystal structure of Arp2/3 complex. Science *294*, 1679-1684.

Biology Open • Supplementary information
